# Supplementary material for: Low complexity RGG-motif sequence is required for Processing body (P-body) disassembly
Source: Nat Commun. 2022 Apr 19;13:2077. doi: 10.1038/s41467-022-29715-5 (PMC9019020; doi:10.1038/s41467-022-29715-5)
Supplement: Supplementary file 1 — Supplementary Information [file 41467_2022_29715_MOESM1_ESM.pdf]

Supplementary Information

Low complexity RGG-motif sequence is required for Processing body (P-body) disassembly

Roy et al. 2022

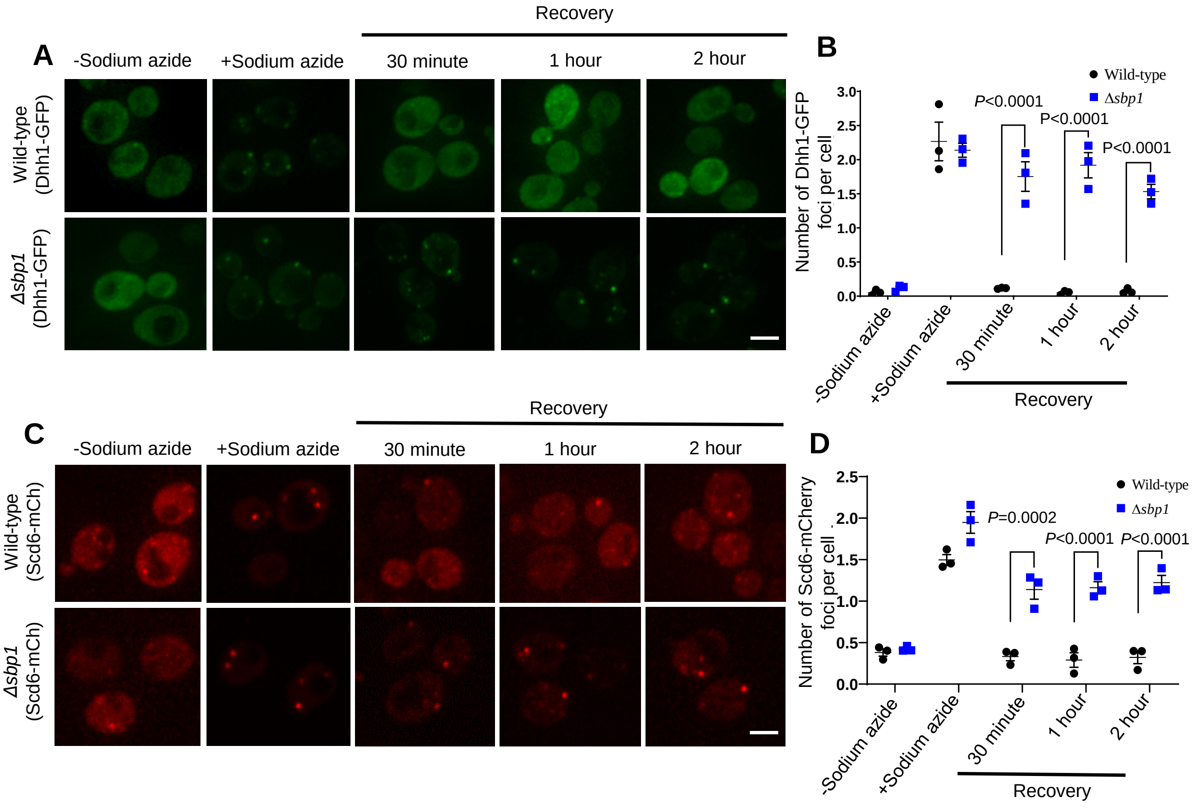

Supplementary Figure 1. PB disassembly is defective in *Δsbp1* strain even after 2 hr recovery. A) Disassembly of Dhh1-GFP granule in wild-type and *Δsbp1* after 30 minutes, 1 hour and 2 hours of extended recovery. Scale Bar, 3µm. B) Quantification of Dhh1-GFP granule per cell in -sodium azide, +sodium azide and extended recovery experiment shown in A. Data plots represent mean ± SEM from of n=3, where 'n' represents number of independent experiments. A two-tailed paired student t-test was used to calculate *P* values. C) Disassembly of Scd6-mCherry granule in wild-type and *Δsbp1* after 30 minutes, 1 hour and 2 hours of extended recovery. Scale Bar, 3µm. D) Quantification of Scd6-mCherry granule per cell in -sodium azide, +sodium azide and extended recovery experiment shown in C. Data plots represent mean ± SEM from of n=3, where 'n' represents number of independent experiments. A two-tailed paired student t-test was used to calculate *P* values.

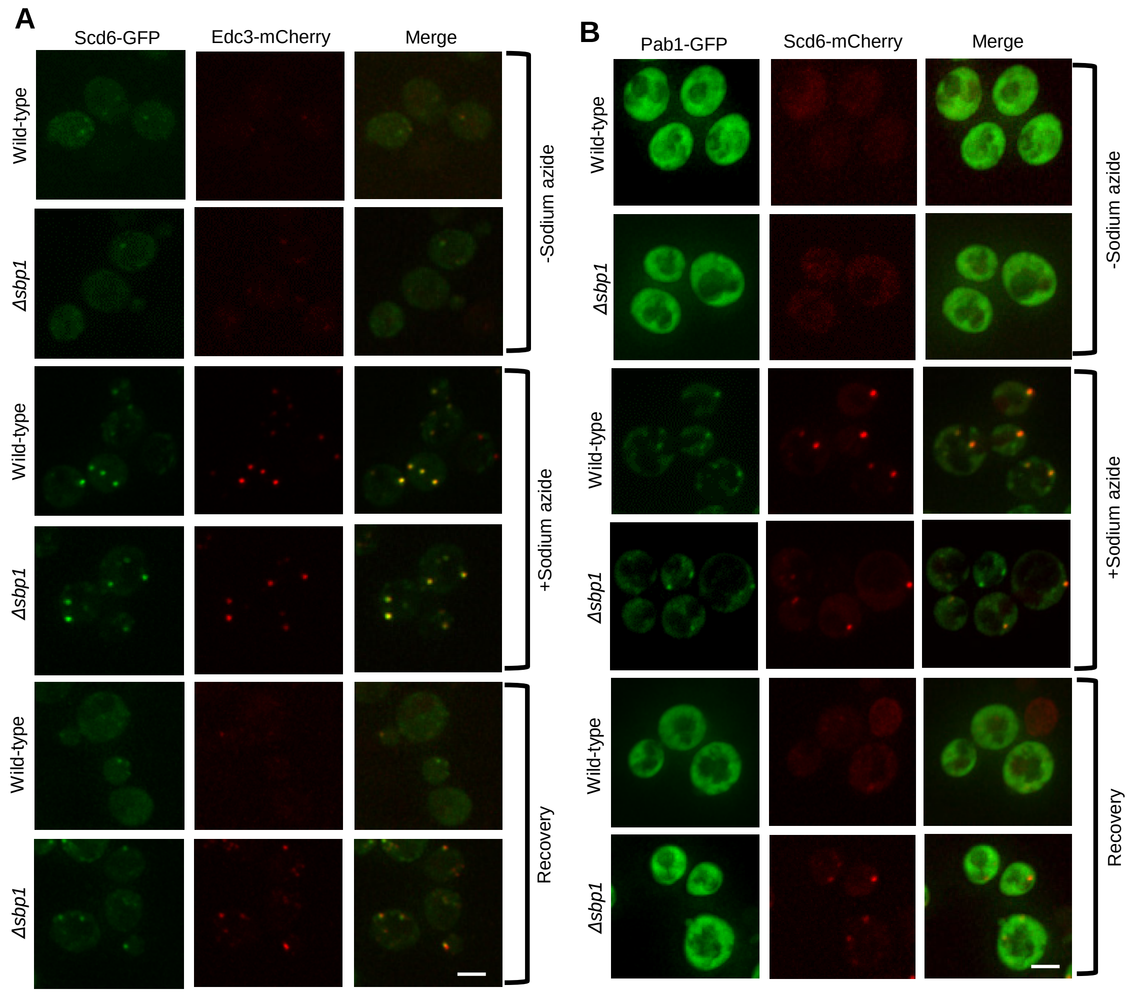

Supplementary Figure 2. Scd6 colocalizes with Edc3 (P-bodies) not Pab1 (stress granule) during recovery. A) Colocalization of Scd6 with Edc3 in wild type and *Δsbp1* during -sodium azide, +sodium azide and recovery conditions. Data conclusions derived from of n=3, where 'n' represents number of independent experiments. Scale Bar, 3µm. B) Colocalization of Scd6 with Pab1 in wild type and *Δsbp1* during -sodium azide, +sodium azide and recovery conditions. Data conclusions derived from of n=3, where 'n' represents number of independent experiments. Scale Bar, 3µm.

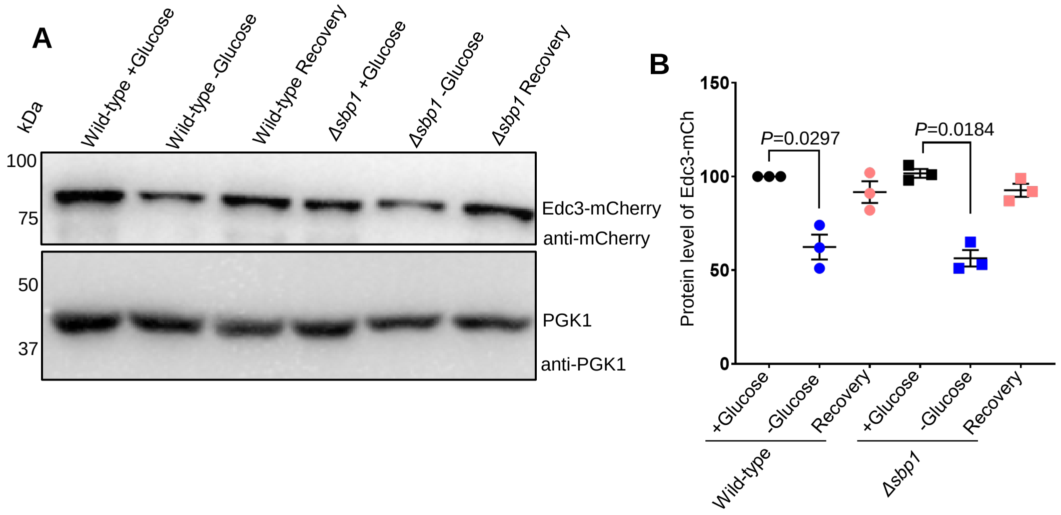

Supplementary Figure 3. Edc3-mCherry granule disassembly defect is not because of change in Edc3 protein levels upon recovery. A) Protein levels of Edc3-mCherry does not change in *Δsbp1* as compared to wild-type during recovery in glucose starvation stress. B) Quantification of Edc3-mCherry protein level from the glucose starvation experiment shown in A. Data plots represent mean ± SEM from of n=3, where 'n' represents number of independent experiments. A two-tailed paired student t-test was used to calculate *P* values.

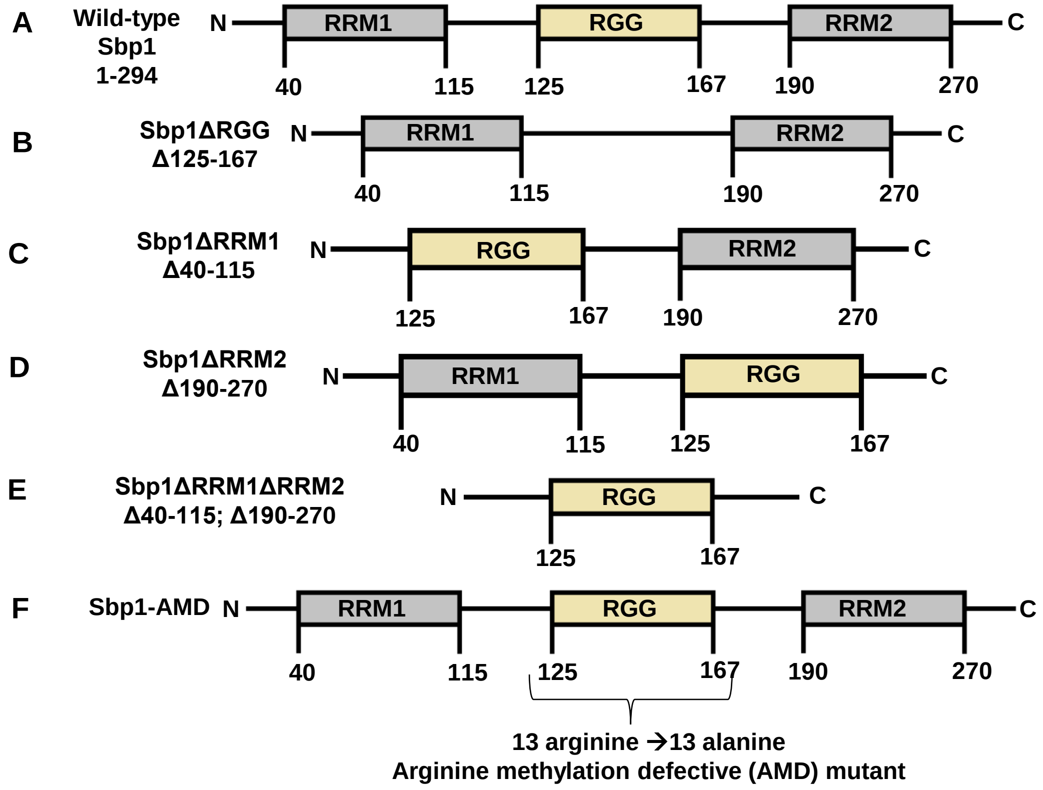

Supplementary Figure 4. Domain organization of Sbp1 depicting the mutants of Sbp1 used in this study. A) Sbp1 contains three functional domains namely N-terminus RRM1, central RGG-motif and C-terminal RRM2 domain. B) The RGG-motif deletion mutant of Sbp1 where the intrinsically disordered residues (125-167) were deleted using site-directed mutagenesis. C) N-terminal RRM1 deletion mutant of Sbp1 (deleted residues 40-115). D) C-terminal RRM2 deletion mutant of Sbp1 (deleted residues 190-270). E) only RGG mutant of Sbp1 where the N-terminus RRM1 (residues 40-115) and C-terminus-RRM2 domains (residues 190-270) were deleted keeping the RGG-motif intact. F) Arginine methylation defective mutant of Sbp1 wherein 13 arginine residues within the RGG-motif were converted to alanine.

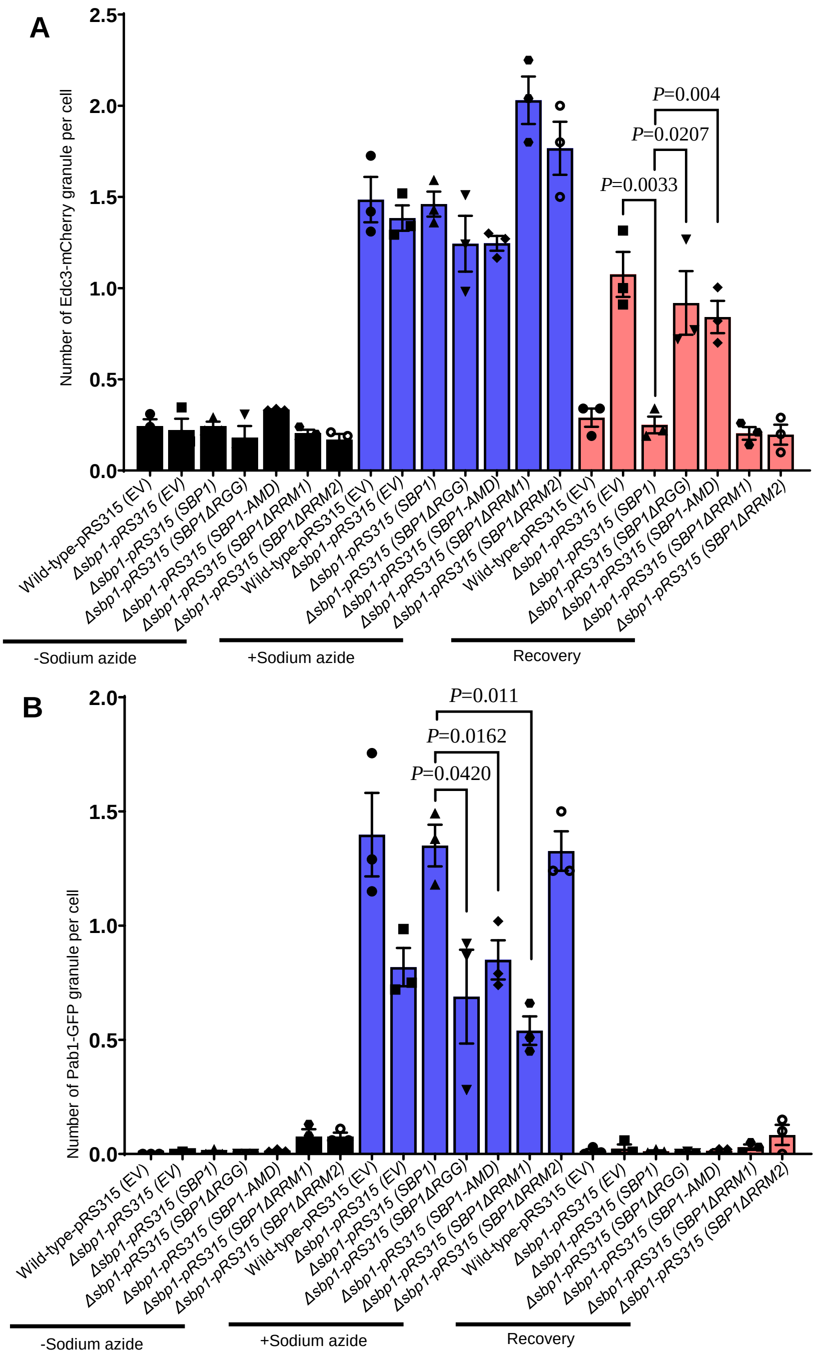

Supplementary Figure 5: Complementation of *Δsbp1* with wild-type *SBP1* and mutants**.** A) Quantitation of Edc3-mCherry granule per cell during -sodium azide, +sodium azide and recovery for the complementation experiment. Plasmid containing wild-type Sbp1 and Sbp1 mutants were transformed in *Δsbp1* cells as indicated. Cells were cultured till 0.35-0.4 OD_{600} and incubated for 30 minutes with or without 0.5% (v/v) sodium azide at 30℃ followed by recovery for 1 hour. Data plots represent mean ± SEM from of n=3, where 'n' represents number of independent experiments. A two-tailed paired student t-test was used to calculate *P* values. B) Quantitation of Pab1-GFP granule per cell during -sodium azide, +sodium azide and recovery for the complementation experiment. Plasmid containing wild-type Sbp1 and Sbp1 mutants were transformed in *Δsbp1* cells as indicated. Cells were cultured till 0.35-0.4 OD_{600} and incubated for 30 minutes with or without 0.5% (v/v) sodium azide at 30℃ followed by recovery for 1 hour. Data plots represent mean ± SEM from of n=3, where 'n' represents number of independent experiments. A two-tailed paired student t-test was used to calculate *P* values.

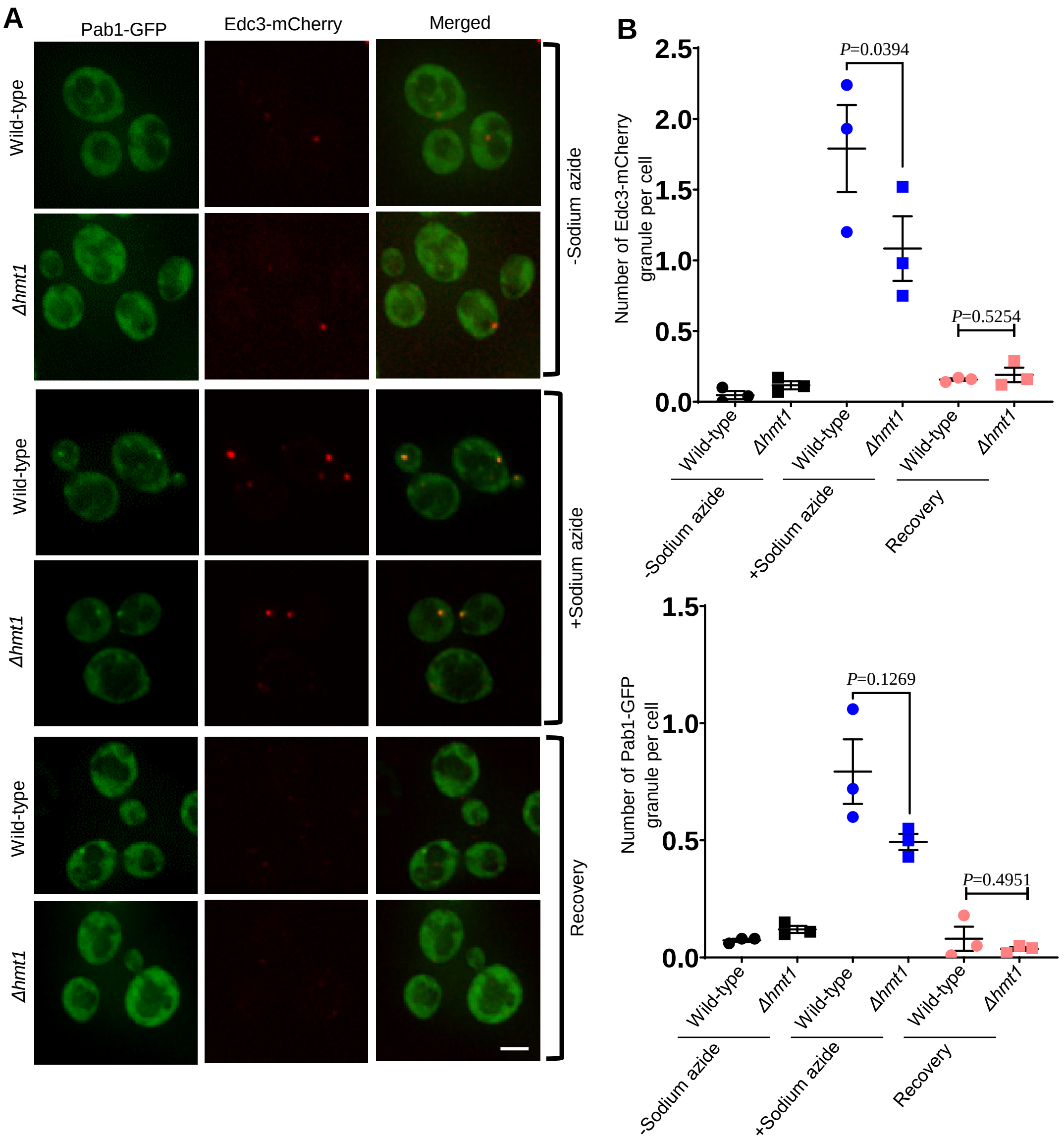

Supplementary Figure 6. Hmt1 deletion does not lead to Edc3 granule disassembly defect. A) *Δhmt1* cells were cultured till 0.35-0.4 OD_{600} and incubated for 30 minutes with or without 0.5% (v/v) sodium azide at 30°C. Subsequently, cells were pelleted by centrifugation (3234 g, 10 seconds, RT) and washed thrice with glucose containing medium. For stress recovery, the resuspended cells were grown for an additional 1 hour at 30°C in media without sodium azide. Scale Bar, 3μm. B) Graph depicting quantitation of SGs (Pab1-GFP) and PBs (Edc3-mCherry) for the experiment presented in A. Data plots represent mean ± SEM from of n=3, where 'n' represents number of independent experiments. A two-tailed paired student t-test was used to calculate *P* values.

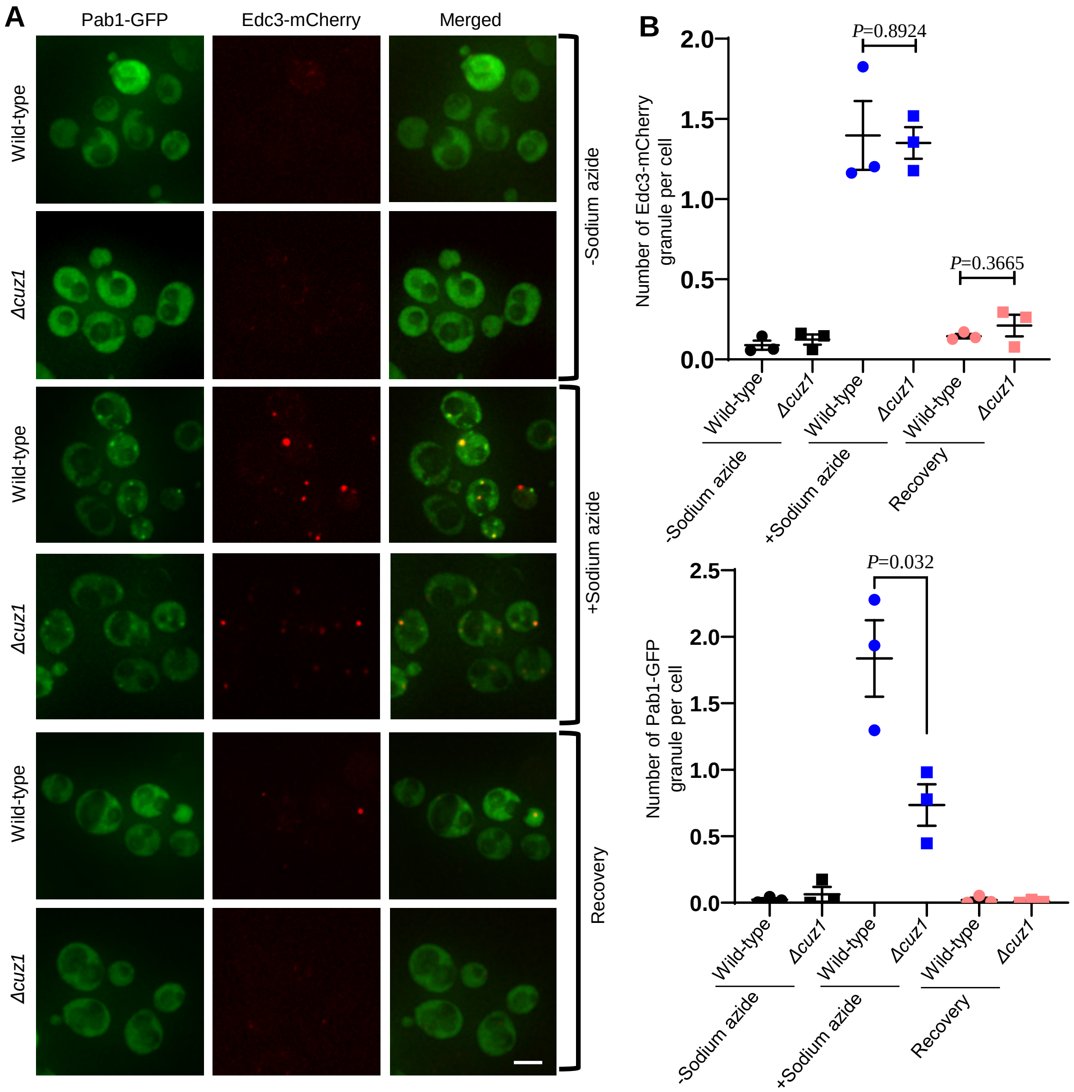

Supplementary Figure 7: Cuz1 deletion does not lead to Edc3 granule disassembly defect. A) *Δcuz1* cells were cultured till 0.35-0.4 OD_{600} and incubated for 30 minutes with or without 0.5% (v/v) sodium azide at 30℃. Subsequently, cells were pelleted by centrifugation (3234 g, 10 seconds, RT) and washed thrice with glucose containing medium. For stress recovery, the resuspended cells were grown for an additional 1 hour at 30℃ in media without sodium azide. Scale Bar, 3µm. B) Graph depicting the number of foci per cell (SGs and PBs individually) in wild-type and *Δcuz1* strain in various culture conditions. Data plots represent mean ± SEM from of n=3, where 'n' represents number of independent experiments. A two-tailed paired student t-test was used to calculate *P* values.

Lab ID	Strain used	Genotype
yPIR1	Wild-type	*MATa his3Δ1 leu2 met15 ura3 ('BY4741')*
yPIR24	Δ*scd6*	*MATa his3Δ1 leu2 ura3 his3 met15 scd6Δ::KanMX*
yPIR25	Δ*sbp1*	*MATa his3Δ1 leu2 ura3 his3 met15 sbp1Δ::KanMX*
yPIR2	Δhmt1	*MATa his3Δ1 leu2Δ0 met15Δ0 ura3Δ0 hmt1Δ::KanMX*
yPIR82	Δ*cuz1*	*MATa his3Δ1 leu2 ura3 his3 met15 cuz1Δ::KanMX*
yPIR89	*EDC3-mCherry*	*MATa his3Δ1 leu2 ura3 his3 met15 EDC3-mCherry::KanMX*
yPIR90	*EDC3-mCherryΔsbp1*	*MATa his3Δ1 leu2 ura3 his3 met15 EDC3-mCherry::KanMX sbp1Δ::HphMX*

Supplementary Table 1: List of strains used in this study

Yeast plasmids

Lab ID	Plasmid used	Composition	Study
pPIR49	EPU	Edc3-mCherry; Pab-GFP; Ura- selection marker; AmpR	Gift from Roy Parker
pPIR20	pPRS315	EMPTY-VECTOR; AmpR	
pPIR24	pPRS315 (*SBP1*)	Sbp1; Leu- selection marker; AmpR	(Bhatter et al., 2019)
pPIR25	pRS315 (*SBP1 ORF ∆RGG*)	Sbp1∆RGG; Leu- selection marker; AmpR	(Bhatter et al., 2019)
pPIR26	pRS315 (*SBP1 ORF AMD*)	Sbp1AMD; Leu- selection marker; AmpR	(Bhatter et al., 2019)
pPIR35	pRS315 (*SBP1 ORF ∆RRM1*)	Sbp1∆RRM1; Leu- selection marker; AmpR	This study
pPIR36	pRS315 (*SBP1 ORF ∆RRM2*)	Sbp1∆RRM2; Leu- selection marker; AmpR	This study
pPIR96	pYES(*SCD6-mCherry*)	expressing Scd6mCherry under its own promoter	(Poornima et al., 2019)
pPIR147	pRS316 (*DHH1-GFP*)	Dhh1-GFP under its own promoter; Ura- selection marker; AmpR	(Mugler et al., 2016)
pPIR21	pRS315 (*SBP1-GFP*)	Sbp1-GFP; Leu- selection marker; AmpR	(Bhatter et al, 2021)
pPIR22	pRS315 (*SBP1∆RGG - GFP*)	Sbp1-GFP∆RGG; Leu- selection marker; AmpR	(Bhatter et al, 2021)
pPIR68	pRS315 (*SBP1∆RRM2 - GFP*)	Sbp1∆RRM2; Leu- selection marker; AmpR	(Bhatter et al, 2021)
pPIR220	pRS315 (*SBP1∆RRM1∆RRM2 - GFP*)	Sbp1∆RRM1∆RRM2; Leu- selection marker; AmpR	(Bhatter et al, 2021)
pPIR295	pAG426 – *Gal-EWSR1-YFP*	EWSR1-YFP; Ura- selection marker; AmpR	(Couthouis et al., 2012)

Bacterial plasmids

pPIR29	His-Sbp1-FLAG	pPROEX-1; AmpR	(Bhatter et al., 2019)
pPIR33	His-Sbp1-FLAG ∆RGG	pPROEX-1; AmpR	(Bhatter et al., 2019)
pPIR249	onlyGST	pGEX-6P-3; AmpR	
pPIR250	His-Edc3-mCherry	pPROEX-1; AmpR	This study
pPIR293	His-mCherry	pPROEX-1; AmpR	This study
pPIR252	LSm-FDF-GST	pGEX-6P-3; AmpR	(Nissan et al., 2010)
pPIR253	FDF-GST	pGEX-6P-3;AmpR	Gift from Roy Parker
pPIR254	YjeF-N-GST	pGEX-6P-3; AmpR	Gift from Roy Parker

Supplementary Table 2: List of plasmids used in this study

Antibody used	Cat ID	Company/Validation
anti-Sbp1 antibody	Home-made	Bhatter et al., 2019
anti-mCherry antibody	ab167453	Abcam; Lattao et al., 2021
anti-GST antibody	2624	CST; Poornima et al., 2016
anti-GFP antibody	902602	BioLegend; Bhatter et al., 2019
anti-PGK1 antibody	Ab113687	Abcam; Poornima et al., 2016
anti-FLAG antibody	F3165	Sigma; Poornima et al., 2016
Goat anti-rabbit secondary antibody	111-035-003	Jackson ImmunoResearch Lab, Bhatter et al., 2019
Goat anti-mouse secondary antibody	115-035-003	Jackson ImmunoResearch Lab, Bhatter et al., 2019

Supplementary Table 3: List of antibodies used in this study

Primer ID	Usage	Sequence (5’->3’)
PIR-GD-mChKan-tag-Edc3-Genome-F	Endogenously tagged Edc3-mCherry using pBS34 as template	TGATCTTTTCGTCACTGACGGGTCCCTGCTATTAGATTTGTCGTACGCTGCAGGTCGAC
PIR-RR-Edc3tagmCherryGenome-R	Endogenously tagged Edc3-mCherry using pBS34 as template	TATACGTATGTATCCAGTTTAGGCTAAAGTAATTCTTGGTGAATTCGAGCTCGTTTAAAC
PIR-B-SBP1-200US-SMAI-S	Creating Δsbp1 in Edc3-mCherry strain from existing Δsbp1 (BY4741) strain	ATTACCCGGGGCAAAACTAGTCGAAAAGCA
PIR-B-SBP1-140DS-SMAI-AS	Creating Δsbp1 in Edc3-mCherry strain from existing Δsbp1 (BY4741) strain	AGCTCCCGGGGCGTCTCATTTTTACATATC
PIR-RR-Edc3mchINFUScloning2-S	Creating Edc3-Cherry in pPROEX-1 bacterial expression plasmid using In-Fusion® HD Cloning Kit (TaKaRa)	TTTTCAGGGCGCCCATATGTCACAATTTGTTGGTTTCGGAG
PIR-RR-Edc3mchINFUScloning2-AS	Creating Edc3-Cherry in pPROEX-1 bacterial expression plasmid using In-Fusion® HD Cloning Kit (TaKaRa)	CAAAACAGCCAAGCTTCTTGTACAGCTCGTCCATGCC
PIR-RR-pPROonlymCherrySDM-S	Creating only-mCherry plasmid from pPROEX-1 (Edc3-mCherry) using site directed mutagenesis	ATTTTCAGGGCGCCCATATGGTGAGCAAGGGC
PIR-RR-pPROonlymCherrySDM-AS	Creating only-mCherry plasmid from pPROEX-1 (Edc3-mCherry) using site directed mutagenesis	GCCCTTGCTCACCATATGGGCGCCCTGAAAAT
PIR-B-Sbp1-500bp-upstream-S	Cloning Sbp1 along with 500bp upstream from start and 140bp downstream from stop codon in pRS315 for complementation experiment.	AGTACCCGGGCTAAGGCAATGTAGGCACTATC
PIR-B-Sbp1-140DS(SmaI)-AS	Cloning Sbp1 along with 500bp upstream from start and 140bp downstream from stop codon in pRS315 for complementation experiment.	AGCTCCCGGGGCGTCTCATTTTTACATATC

Supplementary Table 4: List of primers used in this study
