# Supplementary material for: Low complexity RGG-motif sequence is required for Processing body (P-body) disassembly
Source: Nat Commun. 2022 Apr 19;13:2077. doi: 10.1038/s41467-022-29715-5 (PMC9019020; doi:10.1038/s41467-022-29715-5)
Supplement: Supplementary file 3 — Reporting Summary [file 41467_2022_29715_MOESM3_ESM.pdf]

nature portfolio

Corresponding author(s): PURUSHARTH I RAJYAGURU

Last updated by author(s): Mar 19, 2022

Reporting Summary

Nature Portfolio wishes to improve the reproducibility of the work that we publish. This form provides structure for consistency and transparency in reporting. For further information on Nature Portfolio policies, see our Editorial Policies and the Editorial Policy Checklist.

Statistics

For all statistical analyses, confirm that the following items are present in the figure legend, table legend, main text, or Methods section.

n/a	Confirmed	
☐	☒	The exact sample size (*n*) for each experimental group/condition, given as a discrete number and unit of measurement
☐	☒	A statement on whether measurements were taken from distinct samples or whether the same sample was measured repeatedly
☐	☒	The statistical test(s) used AND whether they are one- or two-sided<br>*Only common tests should be described solely by name; describe more complex techniques in the Methods section.*
☐	☒	A description of all covariates tested
☐	☒	A description of any assumptions or corrections, such as tests of normality and adjustment for multiple comparisons
☐	☒	A full description of the statistical parameters including central tendency (e.g. means) or other basic estimates (e.g. regression coefficient) AND variation (e.g. standard deviation) or associated estimates of uncertainty (e.g. confidence intervals)
☐	☒	For null hypothesis testing, the test statistic (e.g. *F*, *t*, *r*) with confidence intervals, effect sizes, degrees of freedom and *P* value noted<br>*Give P values as exact values whenever suitable.*
☒	☐	For Bayesian analysis, information on the choice of priors and Markov chain Monte Carlo settings
☒	☐	For hierarchical and complex designs, identification of the appropriate level for tests and full reporting of outcomes
☒	☐	Estimates of effect sizes (e.g. Cohen's *d*, Pearson's *r*), indicating how they were calculated

Our web collection on statistics for biologists contains articles on many of the points above.

Software and code

Policy information about availability of computer code

Data collection	SoftWorx version 6.1.3 software installed in DeltaVision Microscope, BioRad Imagelab version 6.0.1 used for acquiring western blot data
Data analysis	ImageJ version 1.53f51, ImageLab version 6.0.1 and GraphPad Prism 4 were used for data analysis

For manuscripts utilizing custom algorithms or software that are central to the research but not yet described in published literature, software must be made available to editors and reviewers. We strongly encourage code deposition in a community repository (e.g. GitHub). See the Nature Portfolio guidelines for submitting code & software for further information.

Data

Policy information about availability of data

All manuscripts must include a data availability statement. This statement should provide the following information, where applicable:

- Accession codes, unique identifiers, or web links for publicly available datasets
- A description of any restrictions on data availability
- For clinical datasets or third party data, please ensure that the statement adheres to our policy

All data is included in the manuscript or accompanying source data file.

Field-specific reporting

Please select the one below that is the best fit for your research. If you are not sure, read the appropriate sections before making your selection.

☒ Life sciences ☐ Behavioural & social sciences ☐ Ecological, evolutionary & environmental sciences

For a reference copy of the document with all sections, see nature.com/documents/nr-reporting-summary-flat.pdf

Life sciences study design

All studies must disclose on these points even when the disclosure is negative.

Sample size	We did not use a statistical test to determine sample size. We adhered to sample sizes reported earlier in studies focusing on characterizing RNA granules in yeast for example, Buchan et al, Cell, 2013).
Data exclusions	No data was excluded
Replication	We have used three or more independent transformants (indicated in Figure legends) for all of our experiments. The quantitation data for all these experiments has been provided. In all cases, results were replicated.
Randomization	No randomization has been performed, as this was not a case/control study including individual participants. Samples were derived form genetically identical, single colonies.
Blinding	Authors performed imaging studies and collected data themselves and hence blinding was not possible. In order to eliminate bias in selection of images, cells were identified for imaging by light microscopy, followed by visualization of these cells by fluorescence microscopy.

Reporting for specific materials, systems and methods

We require information from authors about some types of materials, experimental systems and methods used in many studies. Here, indicate whether each material, system or method listed is relevant to your study. If you are not sure if a list item applies to your research, read the appropriate section before selecting a response.

Materials & experimental systems

n/a	Involved in the study	
☐	☒	Antibodies
☒	☐	Eukaryotic cell lines
☒	☐	Palaeontology and archaeology
☒	☐	Animals and other organisms
☒	☐	Human research participants
☒	☐	Clinical data
☒	☐	Dual use research of concern

Methods

n/a	Involved in the study	
☒	☐	ChIP-seq
☒	☐	Flow cytometry
☒	☐	MRI-based neuroimaging

Antibodies

Antibodies used

The following antibodies were used:
Rabbit anti-mCherry (Abcam, Cat: ab167453)
Rabbit anti-Sbp1 (Home-made, Bhatter et al., 2019)
Mouse anti-GFP (BioLogend, Cat: 902602)
Mouse anti-GST (CST, Cat: 2624)
Mouse anti-Pgk1 (Abcam, Cat: Ab113687)
Mouse anti-FLAG (Sigma, catalogue no. F3165)
Goat anti-rabbit secondary antibody (Jackson ImmunoResearch Lab; Cat: 111-035-003)
Goat anti-mouse secondary antibody (Jackson ImmunoResearch Lab; Cat: 115-035-003)

Validation

These antibodies are all widely used for similar experiments in the field.
Rabbit anti-mCherry (Abcam; Lattao et al., 2021)
Rabbit anti-Sbp1 (Bhatter et al., 2019)
Mouse anti-GFP (BioLegend; Bhatter et al., 2019)
Mouse anti-GST (CST; Poornima et al., 2016)
Mouse anti-Pgk1 (Abcam; Poornima et al., 2016)
Mouse anti-FLAG (Sigma; Poornima et al., 2016)
Goat anti-rabbit secondary antibody (Jackson ImmunoResearch Lab, Bhatter et al., 2019)
Goat anti-mouse secondary antibody (Jackson ImmunoResearch Lab, Bhatter et al., 2019)
